# Potential of Cameroonian isolates of *Beauveria bassiana* and *Metarhizium anisopliae* for the biocontrol of the banana aphid, *Pentalonia nigronervosa*, vector of banana bunchy top virus

**DOI:** 10.1371/journal.pone.0310746

**Published:** 2024-11-07

**Authors:** Aime Cheoh Enoh, Jane-Francis Akoachere, Tatsinkou Bertrand Fossi, Gertrude Membang, Sergine Ngatat, Apollin Fotso Kuate, Rachid Hanna, P. Lava Kumar, Komi K. Mokpokpo Fiaboe

**Affiliations:** 1 International Institute of Tropical Agriculture, Yaoundé, Cameroon; 2 Departments of Microbiology and Parasitology, University of Buea, Buea, Cameroon; 3 Faculty of Agronomy and Agricultural Sciences, University of Dschang, Dschang, Cameroon; 4 Center for Tropical Research, Institute of the Environment and Sustainability, University of California, Los Angeles, CA, United States of America; 5 International Institute of Tropical Agriculture, Ibadan, Nigeria; Government College University Faisalabad, PAKISTAN

## Abstract

*Metarhizium anisopliae* (Metschnikoff) Sorokin and *Beauveria bassiana* (Balsamo) Vuillemin are entomopathogenic fungi commonly used in microbial control of arthropods. In this study, we evaluated the insecticidal potential of six isolates of *B*. *bassiana* (BIITAC10.3.3, BIITAC6.2.2, and BIITAC8.1.5) and *M*. *anisopliae* (MIITAC11.3.4, MIITAC6.2.2, and MIITAC6.4.2) from Cameroon, against the banana aphid *Pentalonia nigronervosa* Coquerel, the vector of the banana bunchy top virus (BBTV). Pathogenicity tests were initially conducted using *B*. *bassiana* and *M*. *anisopliae* isolates at a concentration of 3.2 × 10^6^ conidia/ml on *P*. *nigronervosa* adults sourced from four agroecologies in Cameroon. Four isolates (BIITAC6.2.2, BIITAC10.3.3, BIITAC8.1.5, and MIITAC6.2.2) were highly pathogenic, causing greater than 75% aphid mortality in all populations. A significant decrease in aphid fecundity was observed with BIITAC6.2.2, MIITAC6.2.2, and BIITAC10.3.3. These three isolates were in a test of a series of four fungal concentrations (3.2 × 10^1^, 3.2 × 10^2^, 3.2 × 10^4^, and 3.2 × 10^6^ conidia/ml). produced LC_50_ of 1.31 × 10^1^ and 3.12 × 10^−2^ for BIITAC10.3.3 and MIITAC6.2.2, respectively. MIITAC6.2.2 had the lowest LC_90_ (1.55 × 10^3^). Our results strongly support the continued development of biopesticides based on one or more of the three fungal entomopathogens for the control of banana aphids as a component of an Integrated Pest Management (IPM) strategy for the reduction of the prevalence and transmission of BBTV under field conditions.

## Introduction

Bananas (*Musa* spp.), including plantains, are key food crops that provide nourishment for more than 500 million people worldwide [1]. It is the world’s top fruit crop and a staple food in many tropical countries [2]. Banana productivity is lowest in sub-Saharan Africa (SSA) due to pests and diseases, drought, soil fertility, and low adoption of improved crop management practices [3, 4].

The banana bunchy top disease (BBTD) caused by the banana bunchy top virus (BBTV), a member of the genus Babuvirus and family Nanoviridae is an economically important disease for bananas in Asia, Africa, and the South Pacific regions [4–7].

BBTD can cause a 90–100% reduction in fruit yield within two consecutive seasons [7]. BBTV is spread through infected banana propagules [8] and the banana aphid *Pentalonia nigronervosa* Coquerel (Hemiptera: Aphididae).Banana aphid transmits BBTV in a persistent and non-propagative manner [9, 10].

According to Hu et al. [9], *P*. *nigronervosa* takes about 4 hours of virus acquisition access period (AAP) and 15 minutes of inoculation access period (IAP) to transmit the virus. The transmission efficacy of BBTV was found to be high when aphids were fed on host plants with high virus titer, with an increased aphid population of viruliferous aphids, and with an increase in AAP and IAP [10, 11]. The banana aphid can act as a primary source of spread by introducing viruses into a new field and also plays a major role in the secondary spread of the virus [12]. The epidemiology of the disease is related to banana aphid ecology [13, 14]. The banana aphid has a pantropical distribution and is known to occur in all banana growing areas globally [14, 15]. The aphid is known to occur most commonly on members of the *Musa* spp., which is considered the main host. Occasional occurrences of banana aphids have also been reported on plants from families, Zingiberaceae, Araceae, Cannaceae, and Heliconiaceae [6].

BBTD is difficult to control due to the lack of durable resistance in landraces and improved hybrids. Host plant resistance is considered the most effective strategy for managing plant viral diseases [14]. Considerable variability has been observed in the abundance of banana aphids on various *Musa* genotypes [16–18], but a high level of resistance to the aphid or the virus has not been identified.

Besides the lack of host plant resistance, effective biological control with parasitoids, pathogens, or generalist predators has not been demonstrated for banana aphids. Numerous generalist predators have been found in association with banana aphids, including earwigs (Dermaptera), spiders (Aranea), hoverflies (Syrphidae), and predatory coccinellids (Coccinellidae). However, none of these alone or together have been shown to control aphid populations effectively [19, 20]. Several greenhouse studies have shown that two braconid wasps parasitize banana aphids in the South Pacific [21, 22]. Still, none have been obtained in Africa or South Asia, despite the widespread presence of the same parasitoids [22, 23]. Cecidomyid parasitoid species, *Endaphis fugitiva* **Gagne & Muratori (Diptera: Cecidomyiidae)**, described from Hawaii, was shown to parasitize the aphids [24], but efforts to introduce it into Africa have failed [25].

Banana aphid management using chemical control methods involving the application of organophosphate insecticides like diazinon, imidacloprid, and paraffinic oil is impractical for smallholder farmers in SSA for economic reasons [26]. Both the virus and aphid are managed by destroying virus-infected plants via injection using herbicides and insecticides and replacing them with virus-free plants [27]. Omondi et al. [28] showed that uprooting BBTV-infected plants in managed farms decreased the prevalence of BBTD only by 2%. This was due to the migration of viruliferous banana aphids from diseased plants to infect nearby healthy banana plants. This emphasized the need for banana aphid control to minimize the secondary spread in the managed farms.

Entomopathogenic microorganisms have been promoted, to control the aphid populations, particularly when invertebrate natural enemies are absent or inefficient [29]. *Beauveria bassiana* (Balsamo) Vuillemin (Hypocreales: Cordycipitaceae) and *Metarhizium anisopliae* (Metschnikoff) Sorokin (Hypocreales: Clavicipitaceae) are ubiquitous, anamorphic entomopathogenic fungi that act as natural enemies of aphids and other insects since both fungi have a global distribution [30, 31]. However, the effectiveness of these fungi on banana aphids is relatively scarce. To develop a biopesticide program for several important crop pests in Central Africa, the International Institute of Tropical Agriculture (IITA) station in Yaounde Cameroon collected over 40 *B*. *bassiana* and *M*. *anisopliae* isolates from Central and Southern Cameroon [31].

Three of the isolates of each species in recent evaluation were shown to be highly virulent against cocoa mirid *Sahlbergella singularis* Haglund (Hemiptera: Miridae) [32], *Cosmopolites sordidus* Germar (Coleoptera: Curculionidae) [33]and flea beetle, *Nisotra uniformis* Jacoby (Coleoptera: Chrysomelidae) [34].

This study aimed to assess the effectiveness of three selected isolates each of *B*. *bassiana* (BIITAC10.3.3, BIITAC6.2.2, BIITAC8.1.5) and *M*. *anisopliae* (MIITAC11.3.4, MIITAC6.2.2, MIITAC6.4.2), in controlling banana aphids as part of an integrated strategy to manage the spread of BBTV. Specifically, we sought to determine how the pathogenicity and virulence of the six isolates vary across pathogen species/isolates, and geographic populations of banana aphids selected from several agroecological zones (AEZ) in Cameroon and to rule out that aphids lack resistance to entomopathogenic fungi (EPF) offered by facultative endosymbionts [35, 36].

## Materials and methods

### Aphid collection, rearing, and identification

**Fig 1:**
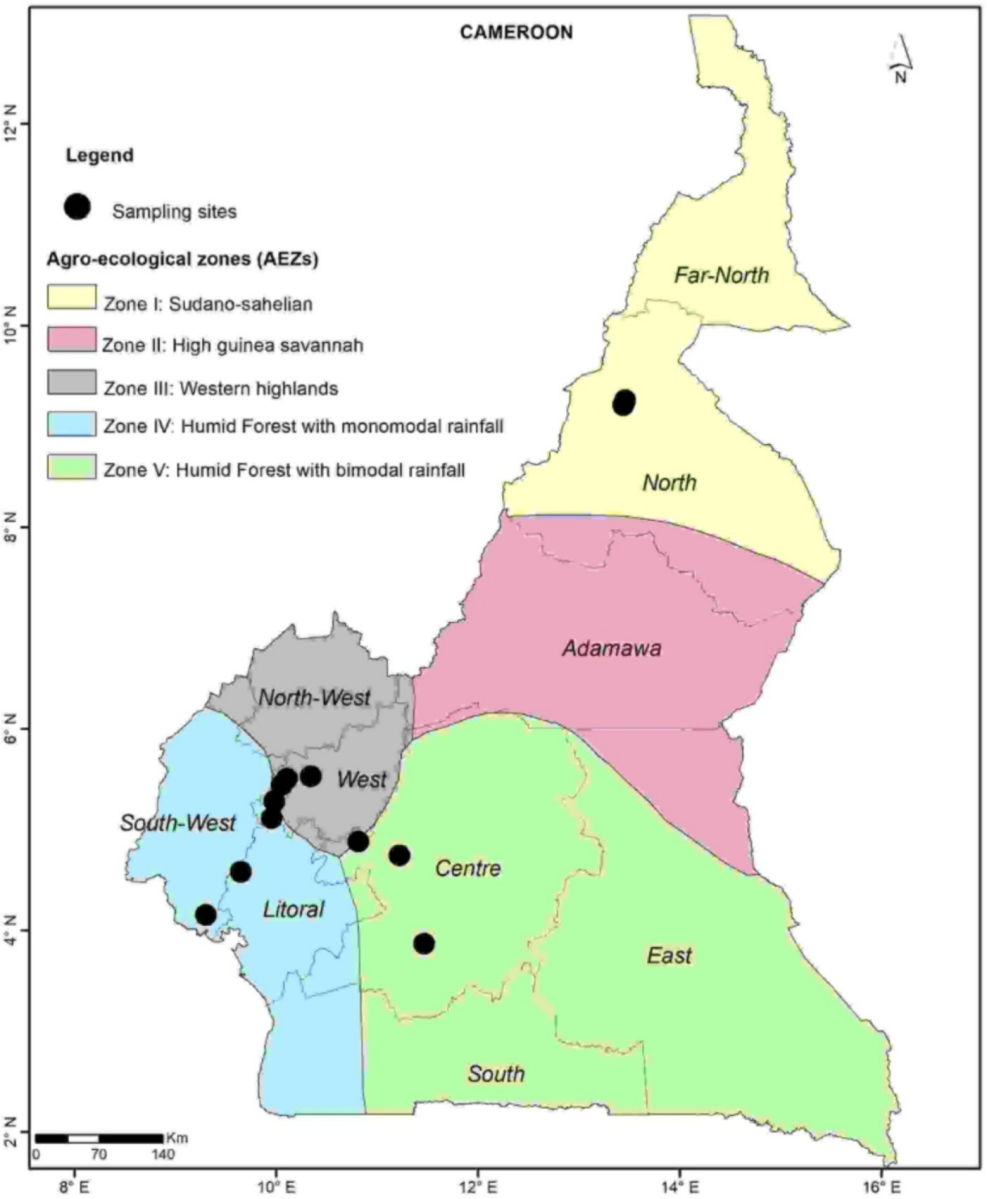
Sampling locations for banana aphids.

*Pentalonia nigronervosa* individuals of all life stages were collected from multiple banana plants in each of 12 locations in four agroecological zones in Cameroon where banana cultivation is common (Fig 1 and S1 Table. Location of aphid collection sites with farm details.). The sampled localities including Sanguere and Mayo Dadi in the Sudano-Sahelian zone (Zone I), Bafou, Bamougoum, Dschang, and Santchou in the Western Highlands (Zone III), Melong, Njombe, and Buea in the Humid Forest with monomodal rainfall (Zone IV), and Bafia, Makenene, and Nkolbisson in the Humid Forest with bimodal rainfall (Zone V).

The main characteristics of the various AEZ were given in S2 Table [37] (S2 Table. Agro-ecological zones (AEZ) and characteristics in Cameroon). The geographic coordinates and altitude of each site were obtained using a GPS recorder (eTrex 30, Garmin International Inc. USA) and the data was used to generate the geographic map using the ArcGIS 10.1.3 software (Fig 1).

In each location, aphids were collected using a fine camel hairbrush from banana plants free of BBTV symptoms [38], The collected aphids were divided into two batches. One batch was placed in 1.5 ml vials containing 90% (v/v) ethanol for species identification using DNA barcoding. The second batch was collected in plastic bowls containing fresh and healthy banana leaf pieces to establish non-viruliferous aphid colonies.

The bowls were sealed with aerated lids and then transported to the laboratory at the IITA in Yaoundé, Cameroon (N03°51′84′′, E11°27′76′′). The aphids were then kept in an insectary at 25 ± 1°C and relative humidity of 70–80% for further maintenance.

Aphids collected from each location were transferred onto healthy, potted tissue-cultured banana plantlets of the plantain variety Essong to maintain the aphid colony. These plantlets were established in insect-proof cages measuring 60cm x 40cm x 50cm in an insect-proof screen house at an ambient temperature with 12 hrs of night and day. The cages had cloth-mesh sides for aeration, and the rest were Plexiglas. The aphids used in this experiment were healthy and did not show signs of fungal infection (mycosis) during maintenance. Appropriate isolation distance between cages was ensured to establish and maintain separate aphid lineages.

To confirm the identity of the aphids, techniques outlined by deWaard et al. [39] were used for DNA extraction and amplification of about 700 bp segment of the mitochondrial cytochrome oxidase subunit 1 (COI) using the primer pairs LepF (5’-ATTCA ACCAATCATAAAGATATTGG-3’) and LepR (5’-TAAACTTCTGGATGTCCAAAAAATC A-3). The thermocycling profile consisted of initial denaturation at 94 ˚C for 1.5 min, followed by 35 cycles of denaturation at 95 ˚C for 30 sec, annealing at 55 ˚C for 1 min, elongation at 72 ˚C for 1.5 min, final elongation at 72 ˚C for 7 min; and a final lap at 4 ˚C [40]. Purified PCR products were sequenced in both orientations using the primers used for PCR by the Sanger sequencing method at the DNA Sequencing Facility at Iowa State University, USA.

### Fungal cultures and conidial suspension preparation

Three *B*. *bassiana* isolates (BIITAC10.3.3, BIITAC6.2.2, BIITAC8.1.5*)* and three *M*. *anisopliae* isolates (MIITAC11.3.4, MIITAC6.2.2, MIITAC6.4.2) were obtained in the form of conidia from the IITA-Cameroon fungal germplasm collection and they were cultured on Potato Dextrose Agar (PDA) in Petri dishes at 25 ± 1 ºC in the dark and 70–80% RH. Conidial suspensions of each isolate were prepared from 14-day-old cultures using sterile distilled water containing 0.1% (v/v) Tween-80. Conidial concentrations were determined using a hemocytometer (Malassez counting chamber) under the microscope (Leica) (40*×*), and different concentrations were prepared through serial dilutions to obtain 3.2×10^1^, 3.2×10^2^, 3.2×10^4^, and 3.2×10^6^ conidia/ml [33]. The conidial solution for each fungal isolate was freshly prepared before use.

### Conidial viability assessment

Conidial suspensions, prepared as described above, were used to test conidia viability. A light microscope (LEICA DMLS, Leica Microsystems GmbH, Wetzlar, Germany) was used to adjust the conidia concentration to 3.2*×*10^6^ conidia/ml, and 100 μl suspension was spread on PDA media in 90-mm Petri dishes. Each isolate was tested in five replicates. Conidial germination was halted after 24 hours by spreading 100 μl of 2% formol on the agar surface in each Petri dish. Each PDA culture plate was sectioned into four sections, and percentage conidial viability was measured by a random assessment of 100 conidia from each of the four sections (400 conidia per plate). Conidia were considered to have germinated if their germ tube was longer than the propagule diameter [41, 42].

### Bioassay procedure and virulence against aphids

Adult apterous aphids (9–12 days old) were allowed to settle for 24 hours on a fresh plantain (Essong) pseudostem piece that had been disinfected in a 1% (v/v) sodium hypochlorite solution and rinsed several times in sterile distilled water before the bioassay. Fresh pseudostems of the Essong plantain were harvested from the IITA Cameroon experimental farms (about 8–12 months old) and chopped to dimensions of 12 *×* 7 mm (L × W). This setup was carried out in plastic boxes (5 *×* 13 *×* 20 cm) covered with lids made of white mousseline sheets to enable aeration. A Pulmic Raptor 1 Garden sprayer (1 liter) that expels approximately 1 ml of the inoculum with one squeeze was used to spray 5 ml of the suspension on aphids. Initially, adult aphids from the 12 populations were sprayed using a single concentration of 3.2 × 10^6^ conidia/ml. All treatments were replicated five times, and 20 adult aphids were used per replicate. Mycosis and mortality were recorded daily for 10 days post-treatment. All dead insects during the 10 days of observation were transferred to Petri dishes lined with sterile cotton and moist filter paper and kept in the dark environment at 25 ± 1°C and 70–80% RH to allow fungal outgrowth. The development of mycosis on dead insects was checked seven days after incubation.

### Concentration-response

The concentration-response bioassays were conducted using only the Bafia aphid population because of its high population density at the time of the experiment and the three best-performing isolates (BIITAC10.3.3, BIITAC6.2.2, and MIITAC6.2.2). Bioassays consisted of spraying banana aphids with a conidial suspension of each isolate at four concentrations (3.2×10^1^, 3.2×10^2^, 3.2×10^4^, and 3.2×10^6^ conidia/ml) *[42]*, and data on aphid morbidity and mortality was collected following the methods described in the previous section. A sterile solution of distilled water with 0.1% (v/v) Tween 80 was administered to the control group. The presence of the fungus on treated aphids was confirmed under a light microscope. To evaluate the fecundity of treated aphids, newly deposited 1^st^ instar nymphs were counted and removed from experimental boxes.

### Statistical analysis

Data obtained from the experiment were analyzed with R software version v4.3.0. *[43]*. Mortality rates from different populations in each agroecological zone were pooled, and cumulative mortality rates were corrected using Abbott’s formula [44]. The Kruskal-Wallis test was used to compare conidial viability, mortality, and mycosis between isolates and agroecological zones. Means were separated using the post hoc Nemenyi test for conidia viability and the Dunn test for mortality and mycosis. LT_50_, LT_90_, LC_50_, and LC_90_ of the isolates were analyzed at 95% confidence limits (CL) using probit analysis with the package "ecotox" with the same version of R software [45]. Corrected mortality and mycosis were summarized using the Rmisc package [46] implemented in R, while the correlation between conidia viability and mortality was done in Excel.

## Results

### Conidia viability

**Fig 2:**
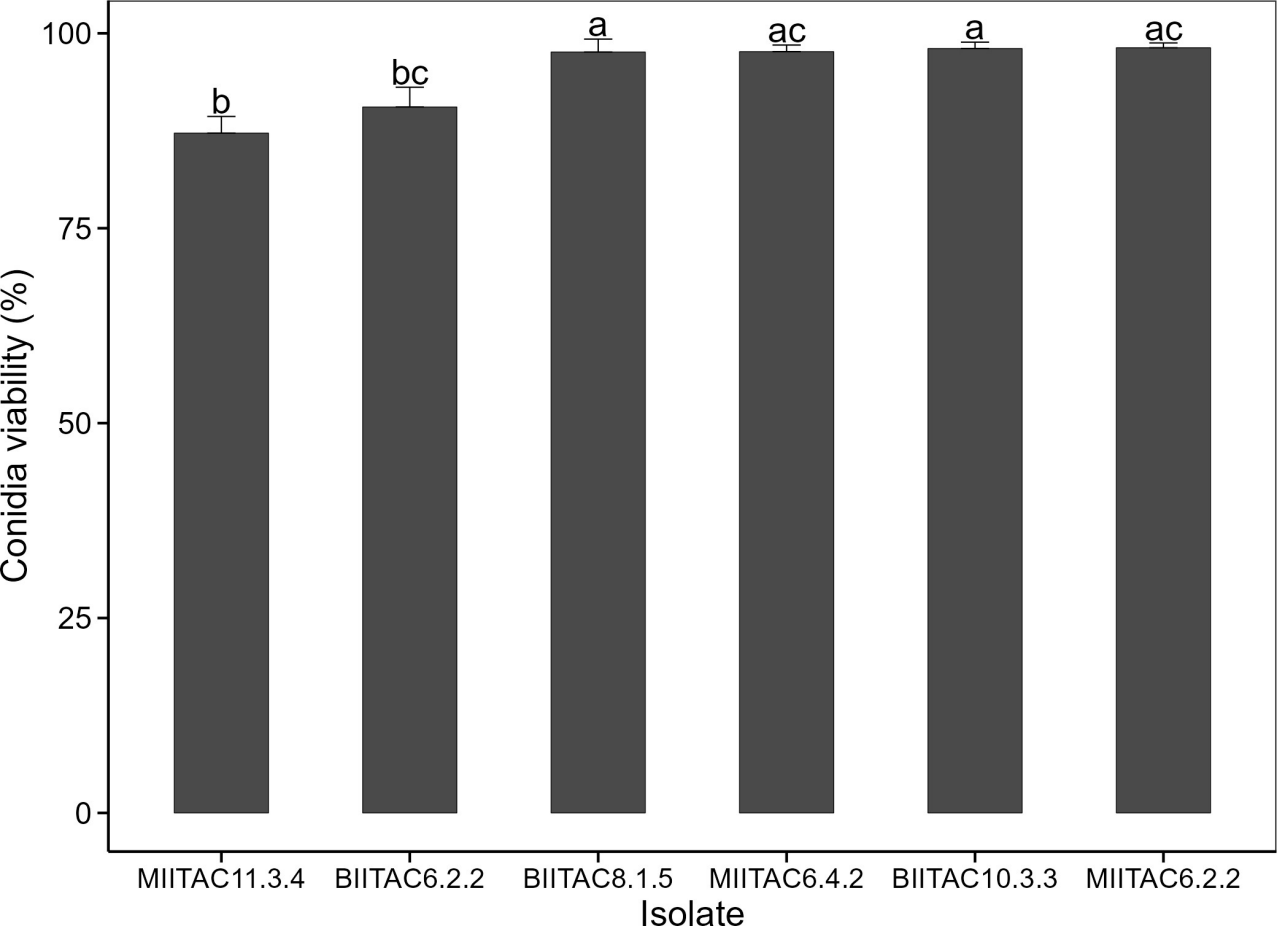
Mean conidial viability of *Beauveria* and *Metarhizium* isolates. Bars with the same letters are not statistically different (Nemenyi post-hoc test, α = 5%).

The mean percentage of conidial viability of the fungal isolates (*Beauveria* and *Metarhizium*) was generally high. BIITAC10.3.3 had the highest germination rate of 98.1% after 24 hours of incubation while MIITAC11.3.4 had the lowest germination rate of 87.2% (Fig 2) (S3 Table. Data for conidial viability). There were significant variations in conidial viability among the six isolates (χ^2^ = 37.49; df = 5; *P < 0*.*001*). BIITAC10.3.3, MIITAC6.2.2, BIITAC8.1.5, and MIITAC6.4.2 had the highest germination rates with similar means, while BIITAC6.2.2 and MIITAC11.3.4 had the least germination rates (Fig 2).

### Susceptibility of banana aphid populations to entomopathogenic fungi at a concentration of 3.2×10^6^

The mortality rates of banana aphid populations from four AEZ exposed to six fungal isolates are presented in Table 1. All isolates were pathogenic to all aphid populations at an initial fungal concentration of 3.2*×*10^6^ conidia/ml, although there were differences in aphid mortality among AEZ (χ^2^ = 8.58; df = 3; *P < 0*.*04*) and isolates (χ^2^ = 146.7; df = 5; *P < 0*.*001*). BIITAC10.3.3, BIITAC6.2.2, BIITAC8.1.5, and MIITAC6.2.2 caused the highest mortality in aphid populations from all AEZ, ranging from 78.6–98.9%. while MIITAC11.3.4 and MIITAC6.4.2 caused the lowest mortality rates, ranging from 57.0–68.9%.

**Table 1:** Percent mortality (mean± SE) of aphid populations from four agroecological zones after ten days of exposure to six entomopathogenic fungal isolates in the laboratory at a concentration of 3.2×10^6^.

Isolate	Zone I	Zone III	Zone IV	Zone V	χ^2^	*P*
BIITAC10.3.3	81.4 ± 4.88bA	86.1 ± 2.89bA	91.3 ± 2.45abA	95.5 ± 1.36aA	9.85	0.02
BIITAC6.2.2	80.0 ± 6.16aA	89.4 ± 3.73aA	85.1 ± 4.08aA	88.8 ± 3.55aA	3.32	0.34
BIITAC8.1.5	89.1 ± 2.55aA	78.9 ± 4.12aA	80.8 ± 3.00aAB	88.1 ± 4.31aA	6.61	0.09
MIITAC11.3.4	62.7 ± 3.53aB	59.0 ± 2.96aB	68.9 ±3.26aBC	62.7 ± 3.34aB	3.57	0.31
MIITAC6.2.2	89.9 ± 4.69abA	78.6 ± 4.35bA	83.9 ± 4.81bA	98.9 ± 0.78aA	16.91	<0.001
MIITAC6.4.2	60.5 ± 2.93aB	58.0 ± 3.41aB	63.7 ± 3.18aC	57.0 ± 4.30aB	2.42	0.49
χ^2^	26.64	49.08	31.26	50.33	-	-
*P*	<0.001	<0.001	<0.001	<0.001	-	-

Means followed by the same lower-case letter within rows or upper -case letter within the column are not significantly different for isolates or populations (Kruskal-Wallis non-parametric and Dunn post-hoc tests, α = 5%). BIITAC = *Beauveria bassiana*; MIITAC = *Metarhizium anisopliae*

Aphid populations from zone V were most susceptible when treated with MIITAC6.2.2 (98.9% mortality) and BIITAC10.3.3 (95.5%) and least susceptible to MIITAC6.4.2 (57.0%). The highest mortality of zone IV aphids was achieved with BIITAC10.3.3 (91.3%). BIITAC6.2.2 caused the highest mortality in zone III (89.4%), while BIITAC8.1.5 and MIITAC6.2.2 caused 89.1 and 89.9% mortalities, respectively, in zone I. The isolates MIITAC6.4.2 and MIITAC11.3.4 caused mortalities below 68.9% of aphids from all AEZ, and notably, they caused the least mortality compared with all other fungal isolates within each agroecological zone.

### Lethal time

Ten days following treatment, BITTAC 10.3.3, BIITAC6.2.2, MIITAC6.2.2, and BIITAC8.1.5 caused the greatest mortality within the least number of days. Generally, these isolates caused 50% (LT_50_) and 90% (LT_90_) mortality of aphid populations across different AEZ, ranging respectively from 3.08 to 5.70 and 8.04 to 15.9 days post-treatment. On the other hand, MIITAC6.4.2, and MIITAC11.3.4 had LT_50_ and LT_90_ values ranging respectively from 4.82 to 7.57 days and 13.5 to 28.2 days (Table 2). Based on the results of pathogenesis assays and the LT_50_ and LT_90_ values, MIITAC6.2.2, BIITAC6.2.2, and BIITAC10.3.3 were selected for the concentration-response bioassays.

**Table 2:** Lethal time (mean LT_50_ and LT_90_ with 95% confidence limits) for aphid populations from different agroecological zones exposed to six entomopathogenic fungal isolates at a concentration of 3.2×10^6^.

Isolates	Agroecological zones							
	I		III		IV		V	
	LT_50_	LT_90_	LT_50_	LT_90_	LT_50_	LT_90_	LT_50_	LT_90_
BIITAC10.3.3	4.46(4.12–4.80)	11.7(10.3–13.6)	4.57(4.38–4.77)	11.2(10.4–12.2)	4.04(3.78–4.31)	10.4(9.42–11.6)	4.55(4.29–4.80)	10.1(9.28–11.1)
BIITAC6.2.2	4.02(3.54–4.50)	12.3(10.3–15.9)	3.67(3.36–3.97)	9.35(8.39–10.7)	3.47(3.13–3.80)	10.6(9.30–12.6)	3.08(2.79–3.37)	9.12(8.13–10.5)
BIITAC8.1.5	4.49(4.11–4.87)	11.3(9.33–13.3)	5.70(5.37–6.04)	14.8(13.1–17.1)	4.82(4.57–5.09)	13.5(12.2–15.2)	5.52(5.19–5.85)	11.9(10.8–13.6)
MIITAC11.3.4	6.87(6.34–7.50)	28.2(22.9–37.0)	6.96(6.63–7.34)	26.0(22.8–30.3)	4.82(4.57–5.09)	13.5(12.2–15.2)	6.32(5.92–6.76)	22.0(18.8–26.8)
MIITAC6.2.2	3.29(2.94–3.36)	8.42(7.44–9.85)	5.15(4.77–5.56)	15.9(13.6–19.4)	3.57(3.10–4.05)	14.4(11.6–19.5)	3.83(3.55–4.09)	8.04(7.39–8.91)
MIITAC 6.4.2	7.11(6.65–7.65)	22.2(18.9–27.4)	7.09(6.71–7.53)	26.0(22.5–30.9)	5.95(5.60–6.33)	23.1(19.9–27.7)	7.57(7.02–8.25)	26.0(21.4–33.3)

BIITAC = *Beauveria bassiana*; MIITAC = *Metarhizium anisopliae*; I, III, IV, V designate respective agroecological zones

### Aphid mycosis

There were no differences across all agroecologies in rates of aphid mycosis caused by BIITAC10.3.3, BIITAC6.2.2, BIITAC8.1.5, and MIITAC 6.4.2, unlike MIITAC11.3.4 and MIITAC6.2.2, which caused significant differences in mycosis among the agroecologies. However, all fungal isolates displayed unique variations within each of the several AEZs (Table 3). Mycosis was not observed in the control group.

**Table 3:** Mycosis rates (mean % ± SE) of aphids exposed to entomopathogenic fungi from four agroecological zones.

Isolate	Zone I	Zone III	Zone IV	Zone V	χ^2^	*P*
BIITAC10.3.3	79.7 ± 4.74aABC	84.1 ± 3.49aAB	80.6 ± 4.36aABC	84.9 ± 2.84aAB	1.34	0.72
BIITAC6.2.2	85.2 ± 6.14aABC	87.4 ± 2.46aA	92.6 ± 1.81aA	91.6 ± 3.02aA	2.69	0.44
BIITAC8.1.5	66.8 ± 7.61aC	76.7 ±3.49aAB	76.4 ± 2.29aC	73.9 ± 3.37aB	1.82	0.61
MIITAC11.3.4	93.6 ± 2.05aA	81.5 ± 3.01abAB	81.0 ± 2.74abBC	72.1 ± 6.34bB	10.3	0.016
MIITAC6.2.2	93.3 ± 2.28abA	782.0 ± 2.85cAB	82.0 ± 3.26bcAB	95.0 ± 1.32aA	16.1	<0.001
MIITAC6.4.2	65.2 ± 7.34aBC	68.9 ± 4.96aB	72.1 ± 4.49aC	82.0 ± 2.80aAB	4.94	0.48
χ^2^	15.73	12.5	21.5	27.3	-	-
*P*	0.008	0.03	<0.001	<0.001	-	-

Means followed by the same lower-case letter within columns or upper-case letter within rows are not significantly different for isolates or populations respectively using Kruskal-Wallis non-parametric and Dunn posthoc tests, α = 5%. BIITAC = *Beauveria bassiana*; MIITAC = *Metarhizium anisopliae*.

### Aphid reproduction

**Fig 3:**
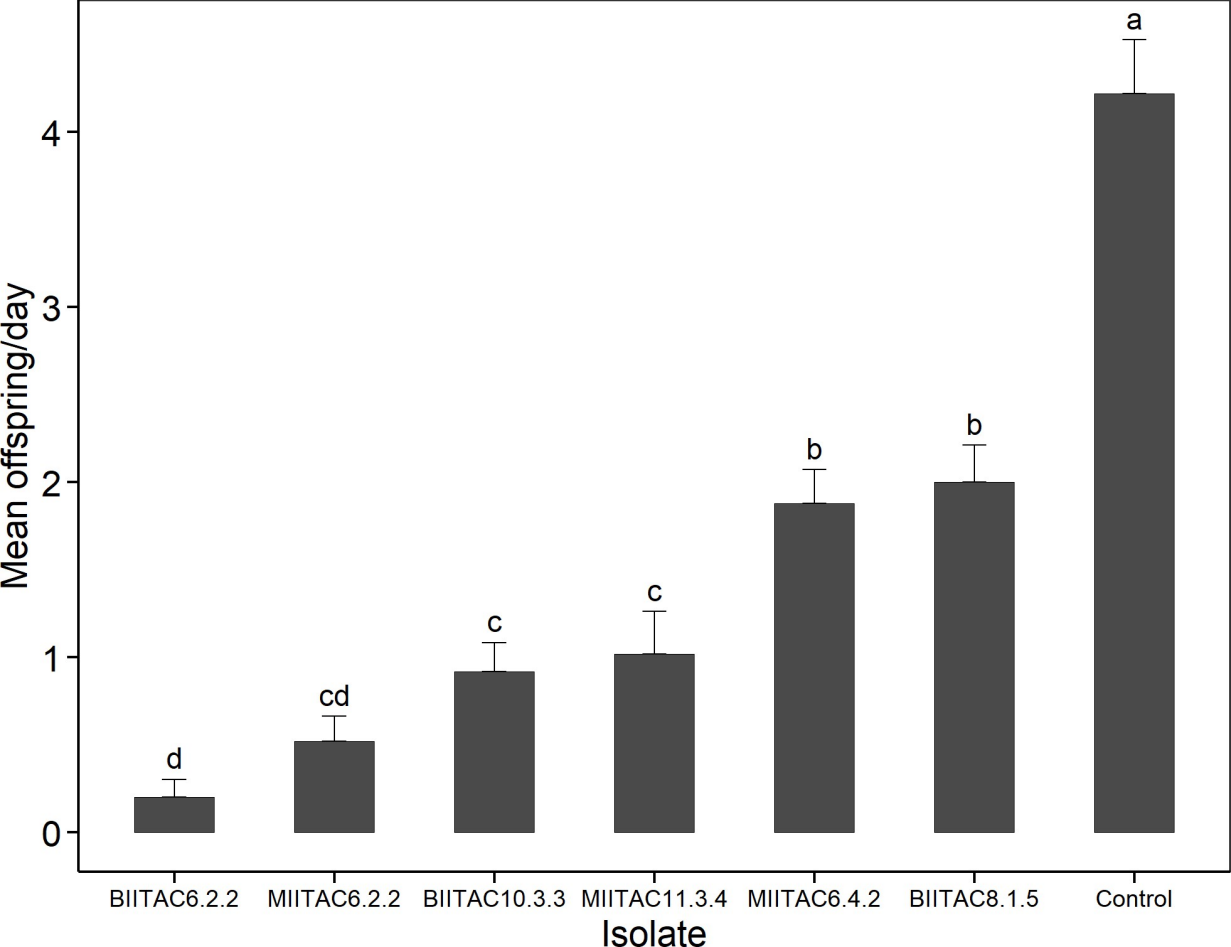
Mean offspring production of the aphid population treated with different fungal isolates at a concentration of 3.2×10^6^.

Aphid reproduction was greatly affected by the different fungal isolates (F = 31.7; df = 6; *P < 0*.*001*) compared with the control group. Moreover, aphids treated with BIITAC6.2.2, MIITAC6.2.2, and BIITAC10.3.3 produced fewer nymphs compared with aphids treated with BIITAC8.1.5, MIITAC11.3.4, and MIITAC6.4.2 (Fig 3).

### Concentration-response

**Fig 4:**
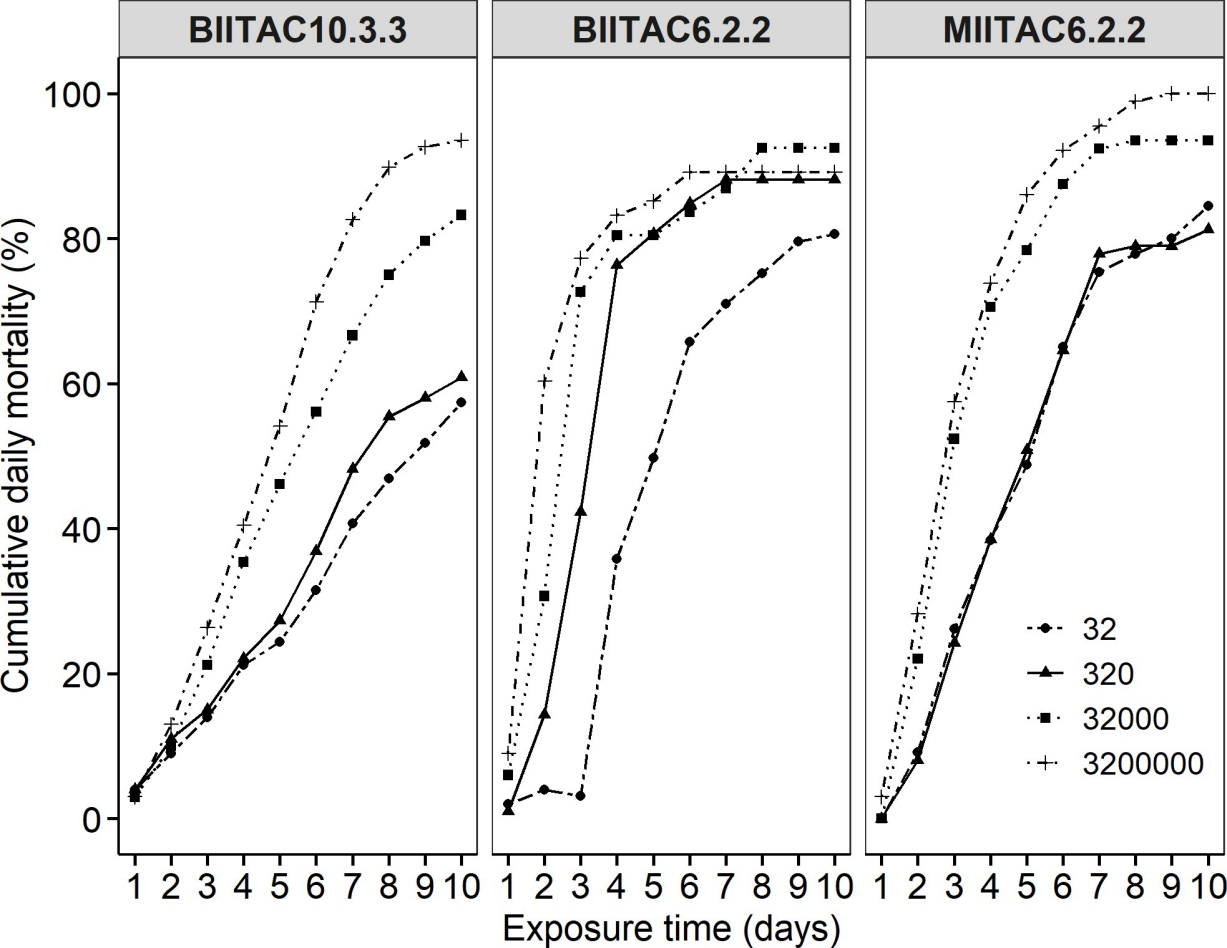
Cumulative mortality rates of aphids treated with four concentrations of BIITAC10.3.3, BIITAC6.2.2, and MIITAC6.2.2.

Conidial concentration of selected fungal isolates significantly affected mortality rates of the aphid population 10 days post-treatment: BIITAC6.2.2 (F = 106.6; df = 3; *P < 0*.*001*), BIITAC10.3.3 (F = 148.1; df = 3; *P < 0*.*001*), and MIITAC6.2.2 (F = 106.9; df = 3; *P < 0*.*001*). Aphid mortality rates increased as the concentration increased (Fig 4). At 5 days post-treatment, the lowest concentration required to eliminate 50% of aphids was from BIITAC6.2.2 (9.34 × 10^−1^ conidia/ml). This was closely followed by MIITAC6.2.2 (7.58 × 10^1^) and then BIITAC10.3.3 (4.32 × 10^5^). BIITAC6.2.2 and MIITAC6.2.2 required lower concentrations of 1.19 ×10^7^ and 1.14 × 10^7^ to cause 90% mortality in the same length of time. Conversely, at 10 days post-treatment, the LC_50_ for MIITAC6.2.2 was lower (3.12 × 10^−2)^ than that of BIITAC10.3.3 (1.31 *×* 10^1^). LC_50_ for BIITAC6.2.2 could not be estimated from our data. Of all isolates, MIITAC6.2.2 had the lowest LT_90_ (1.55 × 10^3^) (Table 4).

**Table 4:** Lethal concentrations (conidia/ml with 95% fiducial limits) of *B*. *bassiana* and *M*. *anisopliae* isolates used against the Bafia banana aphid population at 5 and 10 days after inoculation.

Fungal isolates	LC_50_		LC_90_	
	Day 5	Day10	Day 5	Day10
BIITAC10.3.3	4.32 × 10^5^ (3.51 × 10^4^–1.43 × 10^8^)	1.31 × 10^1^ (8.86 × 10−2–1.22×10^2^)	1.33× 10^13^ (6.11 × 10^9^−9.84×10^23^)	5.4 × 10^5^ (4.66 × 10^4^–1.87 × 10^8^)
BIITAC6.2.2	9.34 × 10^−1^ (1.24 × 10^−23^–9.57 × 10^1^)	*	1.19×10^7^ (9.66 × 10^4^−2.50 × 10^31^)	1.71 × 10^5^ (#)
MIITAC6.2.2	7.58 × 10^1^ (7.95 × 10^0^−3.30 × 10^2^)	3.12 × 10^−2^ (4.29 × 10^−28^–4.99 × 10^0^)	1.14 × 10^7^ (1.12×10^6^−7.43×10^8^)	1.55 × 10^3^ (3.01 × 10^1^–1.53 × 10^7^)

*More than 50% mortality obtained for all concentrations

#fuducial limits could not be estimated.

## Discussion

Entomopathogenic fungi, including *B*. *bassiana* and *M*. *anisopliae*, have been widely studied as biological control agents against various arthropod pests, including aphids [42, 47–49]. Virulence and pathogenicity studies of EPF rarely, however, include multiple populations of a target pest. In this study, we determined the insecticidal potential of Cameroon-indigenous isolates of *B*. *bassiana* and *M*. *anisopliae* against various populations of the banana aphid which were collected from four Cameroon agroecologies that are known to represent much of Central Africa. Preserving spores and maintaining their viability is crucial for long-term storage, research, and applications of EPF. Most studies consider germination rate as a crucial parameter in the selection of microbial agents [50]. In a first step of our study, we determined that conidial viability of our fungal isolates ranged from 87.2 to 98.1%. These results are similar to Membang et al. [33], who found more than 75% viability rates for similar isolates within 24 hours of incubation. The exhibition of such high levels of conidial viability by the EPF isolates is a strong indication that they were suitable for further pathogenicity and virulence studies for the eventual development of a biopesticide for the suppression of the banana aphid.

The results of pathogenicity experiments showed that all tested aphid populations were susceptible to the various fungal isolates but there were considerable differences in susceptibility of various aphid populations to the fungal isolates. For example, BIITAC10.3.3 and MIITAC6.2.2 caused the highest aphid mortality rates of 95.5% and 98.9%, respectively, but the time to reach 50% mortality was the shortest for MIITAC6.2.2 on aphids from AEZ I (LT_50_ = 3.29 days) and BIITAC6.2.2 on aphids from AEZ V (LT_50_ = 3.08 days). There are several possible reasons for the observed differences in susceptibility to EPF among the various geographic populations of the banana aphid used in our study. First, it is widely known that genetic variability of an arthropod can affect its susceptibility to any pathogen. It is therefore, possible that genetic variability among geographic populations of the banana aphid tested in our study could underly differences in their susceptibility to EPF. While genetic variability can be quite large among aphid populations of many species, as in the example of 18 geographic populations of *Myzus persicae* Sulzer (Hemiptera: Aphididae) from Italy [51], the genetic variability of geographic populations of the banana aphid tends to be low, probably due to exclusive asexual reproduction by this aphid [52]. In addition to low genetic variability, the banana aphid is known to harbor the endosymbionts *Buchnera* and *Wolbachia* [53], and possibly other endosymbionts [54], which are known to affect the biology of their arthropod hosts, their relationship with the host plants, and their response to their abiotic environments. It is known that endosymbionts can confer [55] or increase resistance and/or susceptibility to entomopathogenic fungi [56]. Zélé et al. [57], reported that variability in mortality and susceptibility to fungal infections among 12 spider mite populations was related to the presence of endosymbionts generally found in most spider mite species. Different *Wolbachia* strains can either buffer or affect fungal infection depending on the fungal species, host background, or environment. Similarly, *Rickettsia* and *Spiroplasma* conferred resistance to *Pandora neoaphidis* Humber (Entomophthorales: Entomophthoraceae) fungal infections of the pea aphid *Acyrthospihon pisum* (Harris) (Hemiptera: Aphididae) [55]. Paker et al. [56] equally revealed that the bacterial symbiont *Regiella insecticola* Moran (Enterobacterales: Enterobacteriaceae) can defend pea aphids from the fungal entomopathogen *Zoophthora occidentalis* Batko (Entomophthorales: Entomophthoraceae), but did not protect the pea aphid from the generalist fungal pathogen, *B*. *bassiana*. Presently, information on the range and frequency of endosymbionts harbored by the banana aphid populations used in our study is lacking. There is, therefore, a considerable need to identify the endosymbiont profile in banana aphids from various AEZ of Cameroon and elsewhere in Africa and to determine their effects on aphid biology, including its response to infections by entomopathogenic fungi.

Of the six EPF isolates tested in our study, MIITAC11.3.4 and MIITAC6.4.2 were the least pathogenic to the banana aphid, while BIITAC10.3.3, BIITAC6.2.2, BIITAC8.1.5, and MIITAC6.2.2 caused the highest aphid mortality and had the lowest lethal time across the four AEZ. The relatively low aphid mortality caused by MIITAC11.3.4 and MIITAC6.4.2 is similar to that reported by Mahot et al. [32] (56% and 55%, respectively), using the hemipteran host insect *S*. *singularis*. In contrast to our study and that of Mahot et al. [32], Membang et al. [33] and Niyibizi et al. [34] reported higher mortality rates with similar isolates infecting the coleopterans *C*. *sordidus* and *N*. *uniformis*. There are two possible reasons, among others, for the differences among the aforementioned studies. First, it is possible that the *M*. *anisopliae* isolates MIITAC11.3.4 and MIITAC6.4.2 are more pathogenic to coleopterans than hemipteran insects. In contrast, the *B*. *bassiana* isolates BIITAC10.3.3, BIITAC6.2.2, and BIITAC8.1.5 showed comparable aphid mortality rates in our experiment, consistent with the findings of Membang et al. [33] and Niyibizi et al. [34] regarding the coleopterans *C*. *sordidus* and *N*. *uniformis*, respectively. In a comparison of *B*. *bassiana* and *M*. *anisopliae*, Erler et al. [58] found that *M*. *anisopliae* tends to be more pathogenic to soil-dwelling insects, such as *C*. *sordidus* and *N*. *uniformis*, although only the immature stages of *N*. *uniformis* are soil-dwelling. Insects have different structures and defense systems, which can affect fungal infection [59–61]. For example, banana aphids and cocoa mirids are soft-bodied, making it easier for fungal hyphae to penetrate, while banana weevils and flea beetles have a firmer exoskeleton. Moreover, different attributes, such as genetic variation, origin, pathogenicity, and virulence, are used to characterize fungal isolates. These attributes may vary in response to different factors, such as the infected host species, the growth medium, abiotic factors, methods of application and manipulation of the pathogen, and types of formulations used [62].

Second, the differences in mortality in the aforementioned studies could be partly caused by differences in the method of inoculating the insects with the fungi. In our study, fungal suspension was sprayed on the aphid, while Membang et al. [33] and Nyibizi et al. [34] immersed *C*. *sordidus* and *N*. *uniformis* adults in fungal suspension. If that is indeed partly the cause of observed differences in pathogenicity between the three studies, then we would expect differences between our study and that of Mahot et al. [32]; however, that was not the case, as pathogenicity of *Metarhizium* isolates was similar for the banana aphid and *S*. *singularis*, which are both hemipteran insects. They were inoculated using spraying and immersing methods, respectively.

Apart from overall pathogenicity, aphid mortality increased with increasing fungal conidia concentration as demonstrated with the 3 isolates ‐ BIITAC10.3.3, BIITAC6.2.2, and MIITAC6.2.2 ‐ that were tested at four concentrations against a single aphid population (Bafia). Our results are, however, different from other studies using the same isolates against other pests. Treatment with BIITAC10.3.3 had LC_50_ values of 5.09× 10^6^ against banana weevil [33] and 8.97× 10^5^ against cocoa mirid [32]. Mahot et al. [32] reported an LC_50_ value of 4.32×10^6^ for BIITAC6.2.2 tests, while Niyibizi et al. [34] reported an LC_50_ value of 2.00×10^8^ for flea beetles with the same isolate. Treatment with MIITAC6.2.2 led to LC_50_ values of 3.63×10^3^ and 2.18×10^7^ in trials against banana weevil and cocoa mirids, respectively. This shows that BIITAC10.3.3, BIITAC6.2.2, and MIITAC6.2.2 at concentrations of 5.09×10^6^, 2.00×10^8^, and 2.18×10^7^ conidia/ml, respectively, could be used to control all the pests indicated above. Moreover, MIITAC6.2.2 produced 90% (LC_90_) mortality with the lowest concentration (1.55 x 10^3^ conidia/ml) compared to the other two isolates. This indicates that MIITAC6.2.2 can cause high mortality rates at relatively low fungus concentrations, which has economic advantages since it would eventually cost less than the other isolates when used for aphid control.

The fungal isolates used in the study were also shown to produce high (though variable) levels of mycosis in banana aphids, with up to 95% mycosis in certain populations when treated with MIITAC6.2.2. High mycosis levels are highly desirable and entomopathogenic fungi, as sporulating cadavers can become a source of infection, and for the persistence of the fungus in the environment of the targeted arthropods, especially in arthropods, like the banana aphid, where population patches generally contain all aphid life stages, making the entire population vulnerable to fungal infection [63]. High conidia production is essential for the occurrence of horizontal transmission and consequently for inducing epizootics that will contribute to the decline in aphid populations [33, 64]. Moreover, factors like feeding patterns, habitats, host morphology, and pathogen-specific characteristics can all affect the speed of fungal pathogenicity [65].

A significant finding of our study is the reduction in aphid reproduction as a result of their infection with BIITAC6.2.2, MIITAC6.2.2, and BIITAC10.3.3, an effect that can further accelerate the decline in treated aphid populations, in addition to inducing high levels of banana aphid mortality. The fungi may have used insect body resources to produce conidia instead of the host for reproductive output [66].

## Conclusions

Of the six EPF isolates that were used in our study, two *B*. *bassiana* BIITAC6.2.2, BIITAC10.3.3 and one *M*. *anisopliae* MIITAC6.2.2 isolates, together with their induction of high aphid mortality and mycosis and suppression of aphid reproduction show their great potential as biocontrol agents for the management of the banana aphid and banana bunchy top disease. The choice of these three isolates is further bolstered by their relatively low LC_50_ and LT_50_ when used against banana aphids from various agroecologies. The present study demonstrated that significant variability in pathogenicity is observed when identical isolates are utilized to manage aphids from diverse agroecological zones. This underscores the crucial requirement for the screening of IPM tools effective on broad-spectrum host strains and the customization of pest management interventions that are tailored to specific regions, to optimize treatment efficacy.

Further works should consider the development of these isolates as formulated biopesticides and should be tested for their in-field persistence, efficacy at the field level, as well as their pathogenicity to non-target insects. Moreover, our results and those of several other studies with the same isolates support the selection of one or more of the promising EPF isolates for targeting several insect pests in several cropping systems, including bananas, cocoa, and vegetables.

## Supporting information

S1 Table Location of aphid collection sites with farm details. (DOCX)

S2 Table Agro-ecological zones (AEZ) and characteristics in Cameroon. (DOCX)

S3 Table Data for conidial viability. (DOCX)
