# Supplementary material for: Potential of Cameroonian isolates of Beauveria bassiana and Metarhizium anisopliae for the biocontrol of the banana aphid, Pentalonia nigronervosa, vector of banana bunchy top virus
Source: PLoS One. 2024 Nov 7;19(11):e0310746. doi: 10.1371/journal.pone.0310746 (PMC11542849; doi:10.1371/journal.pone.0310746)
Supplement: S1 Table — (DOCX) [file pone.0310746.s001.docx]

**S1 Table .** Location of aphid collection sites with farm details.

| **Agro-ecological zone** | | **Locality** | **Latitude** | **Longitude** | **Croping system** | **Variety** | **Mat age** |
| --- | --- | --- | --- | --- | --- | --- | --- |
| Zone I | Sudano-Sahelian zone | Mayo Dadi-Garoua | 09.20641° | 013.44222° | Near a stream | Unknown | >5years |
|  |  | Sanguere-Garoua | 09.25546° | 013.46149° | Mixed cropping | Unknown | 2-3years |
| Zone III | Western Highlands | Bafou | 05.50423° | 010.10515° | Mixed cropping | Essong | 2-3years |
|  |  | Bamoungoum | 05.52416° | 010.34040° | Mixed cropping | Essong | 2-3years |
|  |  | Sanchou | 05.27775° | 009.97690° | Mixed cropping | Essong | 2-3years |
|  |  | Dschang | 05.43819° | 010.04914° | Mixed cropping | Essong | 2-3years |
| Zone IV | Humid forest (monomodal rainfall) | Buea | 04.15046° | 09.30015° | Mixed cropping | Ebang | 1-2years |
|  |  | Melong | 05.10884° | 009.95185° | Mixed cropping | Essong | 1-2years |
|  |  | Njombe | 04.57878° | 009.64567° | Mixed cropping | Essong | <1years |
| Zone V | Humid Forest (bimodal rainfall) | Bafia | 04.73979° | 011.22177° | Mixed cropping | Ebang | <1years |
|  |  | Nkolbisson | 03.86466° | 011.46539° | Mono cropping | Assung Mbele | >2years |
|  |  | Makenene | 04.87775° | 010.81410° | Mixed cropping | Essong | >3years |
