# Supplementary material for: Potential of Cameroonian isolates of Beauveria bassiana and Metarhizium anisopliae for the biocontrol of the banana aphid, Pentalonia nigronervosa, vector of banana bunchy top virus
Source: PLoS One. 2024 Nov 7;19(11):e0310746. doi: 10.1371/journal.pone.0310746 (PMC11542849; doi:10.1371/journal.pone.0310746)
Supplement: S2 Table — (DOCX) [file pone.0310746.s002.docx]

**S2 Table.** Agro-ecological zones (AEZ) and characteristics in Cameroon [36]

| Agro-ecological zone  (AEZ) | Rainfall  (mm) | Elevation  (m.a.s.l) | Mean annual T°C.(range) |
| --- | --- | --- | --- |
| I. Sudano Sahelian zone | 500-900 | 25-500 | 28 (7.7) |
| III. Western high lands | 1800-2400 | 1500-2500 | 21 (2.2) |
| IV. Humid Forest (monomodal rainfall) | 2000-11000 | 0-2500 | 26 (2.8) |
| V. Humid Forest (monomodal rainfall) | 1500-2000 | 400-1000 | 25 (2.4) |

m.a.s.l = meters above sea level
