# Supplementary material for: Potential of Cameroonian isolates of Beauveria bassiana and Metarhizium anisopliae for the biocontrol of the banana aphid, Pentalonia nigronervosa, vector of banana bunchy top virus
Source: PLoS One. 2024 Nov 7;19(11):e0310746. doi: 10.1371/journal.pone.0310746 (PMC11542849; doi:10.1371/journal.pone.0310746)
Supplement: S3 Table — (DOCX) [file pone.0310746.s003.docx]

**S3 Table.** Data for spore viability

| Fungal isolates | repetition | Ungerminated conidia | Germinated conidia | Total conidia observed | Conidia viability |
| --- | --- | --- | --- | --- | --- |
| BIITAC10.3.3 | 1 | 12 | 88 | 100 | 88 |
| BIITAC10.3.3 | 1 | 0 | 100 | 100 | 100 |
| BIITAC10.3.3 | 1 | 2 | 98 | 100 | 98 |
| BIITAC10.3.3 | 1 | 0 | 100 | 100 | 100 |
| BIITAC10.3.3 | 2 | 0 | 100 | 100 | 100 |
| BIITAC10.3.3 | 2 | 6 | 94 | 100 | 94 |
| BIITAC10.3.3 | 2 | 2 | 98 | 100 | 98 |
| BIITAC10.3.3 | 2 | 0 | 100 | 100 | 100 |
| BIITAC10.3.3 | 3 | 0 | 100 | 100 | 100 |
| BIITAC10.3.3 | 3 | 0 | 100 | 100 | 100 |
| BIITAC10.3.3 | 3 | 0 | 100 | 100 | 100 |
| BIITAC10.3.3 | 3 | 2 | 98 | 100 | 98 |
| BIITAC10.3.3 | 4 | 4 | 96 | 100 | 96 |
| BIITAC10.3.3 | 4 | 0 | 100 | 100 | 100 |
| BIITAC10.3.3 | 4 | 0 | 100 | 100 | 100 |
| BIITAC10.3.3 | 4 | 11 | 89 | 100 | 89 |
| BIITAC10.3.3 | 5 | 0 | 100 | 100 | 100 |
| BIITAC10.3.3 | 5 | 0 | 100 | 100 | 100 |
| BIITAC10.3.3 | 5 | 0 | 100 | 100 | 100 |
| BIITAC10.3.3 | 5 | 0 | 100 | 100 | 100 |
| BIITAC8.1.5 | 1 | 0 | 100 | 100 | 100 |
| BIITAC8.1.5 | 1 | 0 | 100 | 100 | 100 |
| BIITAC8.1.5 | 1 | 2 | 98 | 100 | 98 |
| BIITAC8.1.5 | 1 | 30 | 70 | 100 | 70 |
| BIITAC8.1.5 | 2 | 0 | 100 | 100 | 100 |
| BIITAC8.1.5 | 2 | 16 | 84 | 100 | 84 |
| BIITAC8.1.5 | 2 | 0 | 100 | 100 | 100 |
| BIITAC8.1.5 | 2 | 0 | 100 | 100 | 100 |
| BIITAC8.1.5 | 3 | 0 | 100 | 100 | 100 |
| BIITAC8.1.5 | 3 | 0 | 100 | 100 | 100 |
| BIITAC8.1.5 | 3 | 0 | 100 | 100 | 100 |
| BIITAC8.1.5 | 3 | 0 | 100 | 100 | 100 |
| BIITAC8.1.5 | 4 | 0 | 100 | 100 | 100 |
| BIITAC8.1.5 | 4 | 0 | 100 | 100 | 100 |
| BIITAC8.1.5 | 4 | 0 | 100 | 100 | 100 |
| BIITAC8.1.5 | 4 | 0 | 100 | 100 | 100 |
| BIITAC8.1.5 | 5 | 0 | 100 | 100 | 100 |
| BIITAC8.1.5 | 5 | 0 | 100 | 100 | 100 |
| BIITAC8.1.5 | 5 | 0 | 100 | 100 | 100 |
| BIITAC8.1.5 | 5 | 0 | 100 | 100 | 100 |
| BIITAC6.2.2 | 1 | 16 | 84 | 100 | 84 |
| BIITAC6.2.2 | 1 | 7 | 93 | 100 | 93 |
| BIITAC6.2.2 | 1 | 2 | 98 | 100 | 98 |
| BIITAC6.2.2 | 1 | 14 | 86 | 100 | 86 |
| BIITAC6.2.2 | 2 | 0 | 100 | 100 | 100 |
| BIITAC6.2.2 | 2 | 0 | 100 | 100 | 100 |
| BIITAC6.2.2 | 2 | 8 | 92 | 100 | 92 |
| BIITAC6.2.2 | 2 | 1 | 99 | 100 | 99 |
| BIITAC6.2.2 | 3 | 22 | 78 | 100 | 78 |
| BIITAC6.2.2 | 3 | 38 | 62 | 100 | 62 |
| BIITAC6.2.2 | 3 | 22 | 78 | 100 | 78 |
| BIITAC6.2.2 | 3 | 29 | 71 | 100 | 71 |
| BIITAC6.2.2 | 4 | 4 | 96 | 100 | 96 |
| BIITAC6.2.2 | 4 | 0 | 100 | 100 | 100 |
| BIITAC6.2.2 | 4 | 0 | 100 | 100 | 100 |
| BIITAC6.2.2 | 4 | 0 | 100 | 100 | 100 |
| BIITAC6.2.2 | 5 | 0 | 100 | 100 | 100 |
| BIITAC6.2.2 | 5 | 18 | 82 | 100 | 82 |
| BIITAC6.2.2 | 5 | 0 | 100 | 100 | 100 |
| BIITAC6.2.2 | 5 | 8 | 92 | 100 | 92 |
| MIITAC11.3.4 | 1 | 2 | 98 | 100 | 98 |
| MIITAC11.3.4 | 1 | 23 | 77 | 100 | 77 |
| MIITAC11.3.4 | 1 | 10 | 90 | 100 | 90 |
| MIITAC11.3.4 | 1 | 36 | 64 | 100 | 64 |
| MIITAC11.3.4 | 2 | 12 | 88 | 100 | 88 |
| MIITAC11.3.4 | 2 | 20 | 80 | 100 | 80 |
| MIITAC11.3.4 | 2 | 9 | 91 | 100 | 91 |
| MIITAC11.3.4 | 2 | 4 | 96 | 100 | 96 |
| MIITAC11.3.4 | 3 | 8 | 92 | 100 | 92 |
| MIITAC11.3.4 | 3 | 24 | 76 | 100 | 76 |
| MIITAC11.3.4 | 3 | 16 | 84 | 100 | 84 |
| MIITAC11.3.4 | 3 | 20 | 80 | 100 | 80 |
| MIITAC11.3.4 | 4 | 1 | 99 | 100 | 99 |
| MIITAC11.3.4 | 4 | 0 | 100 | 100 | 100 |
| MIITAC11.3.4 | 4 | 8 | 92 | 100 | 92 |
| MIITAC11.3.4 | 4 | 10 | 90 | 100 | 90 |
| MIITAC11.3.4 | 5 | 15 | 85 | 100 | 85 |
| MIITAC11.3.4 | 5 | 8 | 92 | 100 | 92 |
| MIITAC11.3.4 | 5 | 4 | 96 | 100 | 96 |
| MIITAC11.3.4 | 5 | 26 | 74 | 100 | 74 |
| MIITAC6.4.2 | 1 | 0 | 100 | 100 | 100 |
| MIITAC6.4.2 | 1 | 0 | 100 | 100 | 100 |
| MIITAC6.4.2 | 1 | 0 | 100 | 100 | 100 |
| MIITAC6.4.2 | 1 | 1 | 99 | 100 | 99 |
| MIITAC6.4.2 | 2 | 0 | 100 | 100 | 100 |
| MIITAC6.4.2 | 2 | 8 | 92 | 100 | 92 |
| MIITAC6.4.2 | 2 | 2 | 98 | 100 | 98 |
| MIITAC6.4.2 | 2 | 0 | 100 | 100 | 100 |
| MIITAC6.4.2 | 3 | 0 | 100 | 100 | 100 |
| MIITAC6.4.2 | 3 | 0 | 100 | 100 | 100 |
| MIITAC6.4.2 | 3 | 10 | 90 | 100 | 90 |
| MIITAC6.4.2 | 3 | 0 | 100 | 100 | 100 |
| MIITAC6.4.2 | 4 | 0 | 100 | 100 | 100 |
| MIITAC6.4.2 | 4 | 8 | 92 | 100 | 92 |
| MIITAC6.4.2 | 4 | 10 | 90 | 100 | 90 |
| MIITAC6.4.2 | 4 | 1 | 99 | 100 | 99 |
| MIITAC6.4.2 | 5 | 0 | 100 | 100 | 100 |
| MIITAC6.4.2 | 5 | 0 | 100 | 100 | 100 |
| MIITAC6.4.2 | 5 | 0 | 100 | 100 | 100 |
| MIITAC6.4.2 | 5 | 7 | 93 | 100 | 93 |
| MIITAC6.2.2 | 1 | 9 | 91 | 100 | 91 |
| MIITAC6.2.2 | 1 | 8 | 92 | 100 | 92 |
| MIITAC6.2.2 | 1 | 2 | 98 | 100 | 98 |
| MIITAC6.2.2 | 1 | 4 | 96 | 100 | 96 |
| MIITAC6.2.2 | 2 | 6 | 94 | 100 | 94 |
| MIITAC6.2.2 | 2 | 0 | 100 | 100 | 100 |
| MIITAC6.2.2 | 2 | 0 | 100 | 100 | 100 |
| MIITAC6.2.2 | 2 | 1 | 99 | 100 | 99 |
| MIITAC6.2.2 | 3 | 1 | 99 | 100 | 99 |
| MIITAC6.2.2 | 3 | 2 | 98 | 100 | 98 |
| MIITAC6.2.2 | 3 | 0 | 100 | 100 | 100 |
| MIITAC6.2.2 | 3 | 0 | 100 | 100 | 100 |
| MIITAC6.2.2 | 4 | 0 | 100 | 100 | 100 |
| MIITAC6.2.2 | 4 | 0 | 100 | 100 | 100 |
| MIITAC6.2.2 | 4 | 2 | 98 | 100 | 98 |
| MIITAC6.2.2 | 4 | 1 | 99 | 100 | 99 |
| MIITAC6.2.2 | 5 | 1 | 99 | 100 | 99 |
| MIITAC6.2.2 | 5 | 0 | 100 | 100 | 100 |
| MIITAC6.2.2 | 5 | 0 | 100 | 100 | 100 |
| MIITAC6.2.2 | 5 | 0 | 100 | 100 | 100 |
